# Racial and Age Group Disparities in the Effect of Social Distancing on Health Status

**DOI:** 10.1093/geroni/igab046.749

**Published:** 2021-12-17

**Authors:** Karen Lincoln

**Affiliations:** University of Southern California, Los Angeles, California, United States

## Abstract

African Americans are dying from COVID-19 at younger ages than Whites. Social distancing (SD) prevents the spread of the virus, but because of work demands, transportation needs, and living arrangements SD may be difficult for many African Americans, many of whom are experiencing higher unemployment, poverty, food insufficiency, and social isolation. This study will determine if the health of African Americans and Whites are differentially impacted by SD measures. SD rules can increase or decrease health disparities by: (a) directly impacting the symptoms and progression of chronic health conditions; (b) influencing availability of protective factors and exposure to risk factors; and (c) mitigating or exacerbating the effects of sources of disparities. These hypotheses will be tested using data from the Understanding America’s COVID Study. Findings can advance understanding of how public health requirements can reduce or increase health disparities and identify protective factors to facilitate adherence to public health guidelines.

